# Implantation of polyglycolic acid mesh over lung resection staple lines to prevent air leaks

**DOI:** 10.1016/j.xjtc.2025.05.022

**Published:** 2025-06-11

**Authors:** Lary A. Robinson, Sandra Bryant, Xiaoqi Sun, Mokshitha S. Kaki, Samuel T. Freyaldenhoven, Taylor Schwer, Alexis Bailey, Youngchul Kim

**Affiliations:** a Division of Thoracic Oncology, Moffitt Cancer Center, Tampa, Fla; b Department of Obstetrics and Gynecology, University of South Florida, Tampa, Fla; c Department of Biostatistics and Bioinformatics, Moffitt Cancer Center, Tampa, Fla

**Keywords:** postoperative air leak, chest tube, polyglycolic acid mesh, lung resection, lung cancer surgery, thoracic surgery, alveolar-pleural fistula

## Abstract

**Background:**

Postoperative air leak is the most common complication following lung resection, occurring in 30% to 58% patients. It requires postponing chest tube removal and contributes to postoperative pain, pneumonia, empyemas, and increased hospital length of stay and cost. We placed a double layer of absorbable polyglycolic acid mesh over the parenchymal staple lines at the end of every major lung resection and retrospectively reviewed the results compared to a cohort of similar lung resections without the use of mesh.

**Methods:**

We retrospectively reviewed consecutive patients undergoing segmentectomy, lobectomy, or multilobe lung resection (one resection was lobectomy or segmentectomy) between Novermber 2020 and July 2024 who had placement of a double layer of polyglycolic mesh over parenchymal staple lines held in place with lung sealant. The control cohort comprised consecutive patients undergoing the same resections without the use of mesh during the first 18 months of the study period. Nonparametric statistical tests were used.

**Results:**

A total of 250 patients were analyzed, including 125 with mesh and 125 without mesh. The mesh group comprised 41 lobectomies, 83 segmentectomies, and 25 multilobe procedures, and the no-mesh group included 44 lobectomies, 80 segmentectomies, and 21 multilobe procedures. There were no differences in demographics or comorbidities between the 2 groups except for a higher rate of severe chronic obstructive pulmonary disease in the mesh patients. There were no mortalities, empyemas, or wound infections in either group. Use of the mesh was associated with significantly reduced length of hospital stay in both group (3.1 ± 1.7 days for mesh, 3.6 ± 3.0 days for no mesh; *P* = .028), and was especially effective in multilobe resections.

**Conclusions:**

Placing a double layer of polyglycolic acid mesh over the parenchymal staple lines in major lung resections is a safe, effective adjunct to reduce postoperative air leaks, resulting in a significant decrease in hospital length of stay.

## Graphical Abstract

**Figure:**
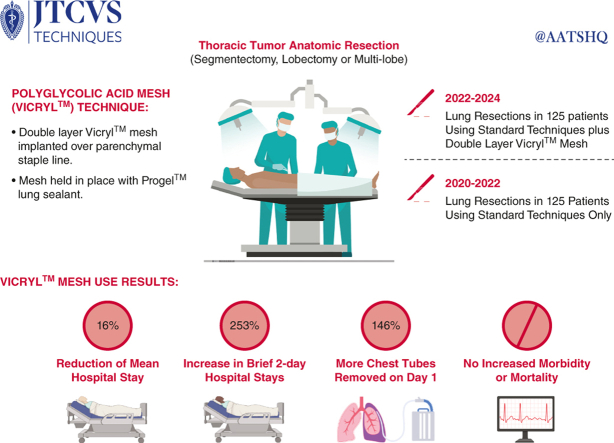

**Figure:**
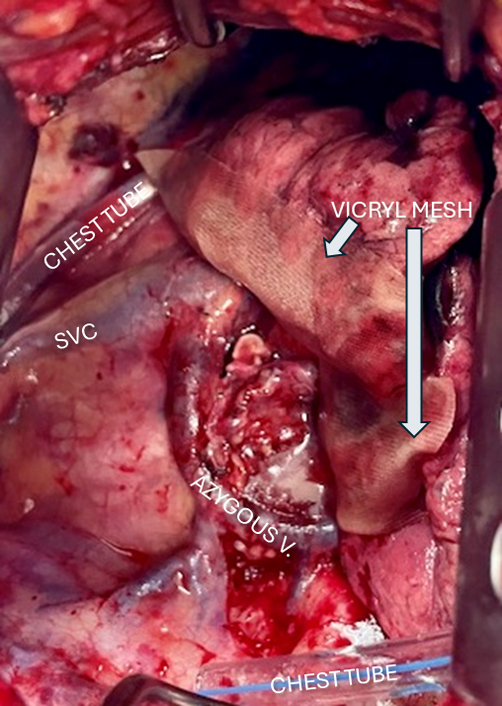
Intraoperative reinforcement of parenchymal staple lines on completion of lung resections with a double layer of polyglycolic mesh is associated with improved pneumostasis and shorter hospital stays.

**Central Message**

Intraoperative reinforcement of parenchymal staple lines on completion of lung resections with a double layer of polyglycolic mesh is associated with improved pneumostasis and shorter hospital stays.

**Perspective**

Despite the use of various published techniques to improve pneumostasis with lung resection, 30% to 58% of patients still have a significant postoperative air leak. Application of a double layer of polyglycolic mesh over parenchymal staple lines held in place by lung sealant is a safe, inexpensive, and effective method to further decrease postoperative air leaks and shorten hospitalizations.

Postoperative air leak is the most common complication after lung resection, occurring in 30% to 58% of patients.^1^^,^^2^ Although most air leaks will close spontaneously, they still require postponing chest tube removal, which contributes to postoperative pain, which can result in pneumonia and even empyema and increases the hospital length of stay. If the leak is severe and prolonged, the air leak may require an intervention, including reoperation.

Various intraoperative and postoperative surgical strategies to prevent air leaks have been proposed.^3^ Increasing the visceral-to-parietal pleural apposition is a key strategy when possible. Without a large postoperative intrapleural air space, air leaks will be less frequent. Despite all the techniques described to prevent air leaks, they remain a frequent occurrence.

Ueda and colleagues,^4^ in a small series of 28 patients, used polyglycolic acid mesh to cover parenchymal suture lines while holding the mesh in place with fibrin glue to successfully stop air leaks in a mean of 1 day, but with a mean hospital length of stay of 7.1 days. We elected to use this technique on a large cohort of lung resection patients, placing a double layer of absorbable polyglycolic acid mesh over the parenchymal staple lines at the end of every major lung resection, to see if this adjunct to surgery would improve pneumostasis. We retrospectively reviewed our results compared to a cohort of similar lung resections in which this technique was not used.

## Methods

### Patient Data

We conducted this retrospective cohort study of consecutive lung resection patients undergoing surgery before and after adopting the techniques of polyglycolic mesh implantation over parenchymal staple lines at Moffitt Cancer Center between November 2020 and June 2024. Approvals were obtained from the Moffitt Scientific Review Committee and Advarra Institutional Review Board (Protocol MCC 22615; approved September 1, 2023).

All patients had undergone a curative resection of a thoracic neoplasm by segmentectomy, lobectomy, or multilobe lung resection (at least one resection was a lobectomy or segmentectomy) by limited thoracotomy between August 2022 and June 2024 by a single surgeon (L.A.R.). The surgeon placed a double-layer absorbable polyglycolic acid mesh (Vicryl polyglactin 910; Ethicon) over parenchymal staple lines held in place with a polyethylene glycol lung sealant (Progel; BD). The results were compared to results seen in a control cohort of the same number of consecutive patients undergoing the same lung resections using the same surgical techniques but without polyglycolic mesh performed by the same surgeon over the prior 18 months (November 2020 to July 2022).

### Surgical Technique

Surgery in both cohorts was performed by a single thoracic surgeon (L.A.R.) using the same standard surgical techniques and perioperative procedures, with the exception of the use of polyglycolic acid mesh in all consecutive patients in the later cohort. The preoperative instructions to both cohorts were uniform and included a 5-day immunonutrition protocol (N-ERAS protocol^5^). The standard anesthesia technique for thoracic surgical cases was general endotracheal anesthesia with intravenous induction, maintenance with volatile anesthetics and intravenous opioids, rigorous normothermia, and 1-lung ventilation via a double-lumen tube. This technique did not change during the 43-month study period. All anatomic lung resections—segmentectomy, lobectomy, and multilobe resections—were performed via an open 12-cm serratus-sparing thoracotomy. Bronchial stumps were covered with a pleural flap or occasionally an intercostal muscle flap. Mediastinal lymphadenectomy was performed in all cases. An apical parietal pleural tent was created in all upper lobectomies and upper lobe segmental resections. An extrapleural pain management catheter (ON-Q) was implanted routinely in all patients. Two 28 Fr chest drainage tubes were placed in all patients, connected to −5 cm H_2_O underwater seal suction (Pleur-evac; Teleflex). Digital system drainage containers were not used.

**Figure 1:**
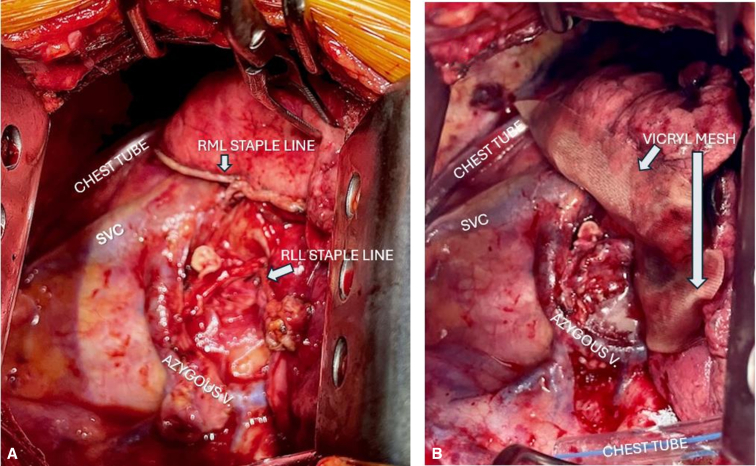
A, View of the right pleural cavity via right thoracotomy after resection of the right upper lobe. B, Same view after placement of double-layer polyglycolic mesh over minor and major fissure staple lines. *RML*, Right middle lobe; *SVC*, superior vena cava; *RLL*, right lower lobe.

In all patients in both cohorts, once the lung resection was completed and prior to chest closure, the pleural cavity was filled with saline. The anesthesiologist then held 30 cm H_2_O positive airway pressure in the endotracheal tube to test the staple lines, which were visually inspected by the surgeon for air leak. Any leak found was suture-repaired with 4-0 Vicryl horizonal mattress, buttressed by small pieces of viable muscle tissue. No blood patches were used. After confirming the absence of air leak, Progel lung sealant (2 vials) was applied in the control, no-mesh cohort. For the mesh cohort, a 3-cm-wide double-layer of saline-moistened polyglycolic acid (Vicryl) mesh was cut to the length of the parenchymal staple line(s) and placed directly over the staple line on the collapsed lung. One vial of Progel lung sealant was sprayed over the mesh to hold it in place. The chest incision was then closed in a routine manner using absorbable polyglycolic acid Vicryl sutures. Figure 1, *A* shows an intraoperative view of the remaining staple lines after resection of the right upper lobe through an right posteriolateral thoracotomy, and Figure 1, *B* shows the staple line after mesh implantation.

All the patients received 1 preoperative 30-mg intravenous dose of methylprednisolone and 1 postoperative dose the evening after surgery. Beginning preoperatively, prophylactic subcutaneous heparin and an intermittent leg compression device were used in all patients. Immediately after surgery, all patients received 1 dose of 0.4 mg/kg desmopressin and a 5-g intravenous bolus of aminocaproic acid (Amicar; Clover Pharmaceuticals) over 1 hour, followed by 1 g/h for 4 hours. The threshold for blood transfusions did not change at any time during the entire study period. Respiratory insufficiency was defined as the inability to ventilate sufficiently with a safe partial pressure of CO_2_ (<55 mm Hg) or could not maintain an oxygen saturation >89% with maximal supplemental oxygen. The decision to use bilevel positive airway pressure or to intubate a patient was made jointly by the surgeon and anesthesiologist or pulmonologist. We recorded a respiratory complication if a patient had required bilevel positive airway pressure or intubation for >1 day. There were no changes in the surgical techniques or the postoperative patient care pathway during the study period.

### Clinical Information

The patients’ medical records were reviewed from the electronic medical record. Comorbidities were compared using the Charlson Comorbidity Index.^6^ Hospital length of stay was recorded as the number of whole days. Hospital discharge criteria were as follows: (1) ambulatory, (2) tolerating a regular diet, (3) bowels moving, (4) pain well-controlled by oral medications, and (5) chest tubes removed or (rarely) the presence of a small air leak with the lung remaining fully expanded without suction with a chest tube and Heimlich valve (Pneumostat; Getinge). The postoperative care pathways for all patients in each group was directed by the primary surgeon (L.A.R.), remained unchanged during the entire study period, and uniformly used the principles of the Kehlet multimodal fast-track program (also known as Enhanced Recovery After Surgery [ERAS]).^7^^,^^8^ These principles included minimizing the overnight fast, promoting early ambulation and nutrition, avoiding the use of tubes, minimizing the use of opioids, and the judiciously using balanced fluids. The criteria for chest tube removal were no air leak for 24 hours with a vigorous cough and <50 mL of pleural fluid drainage per 8 hours.

Postoperatively, all patients were followed as outpatients for a minimum of 6 months until they returned for their 6-months surveillance chest computed tomography scan. Most patients were followed much longer in our Lung Surveillance Clinic.

### Statistical Consideration

Descriptive statistics were calculated, including frequency and percentage for categorical variables and mean (standard deviation) or median (range) for continuous variables. A propensity score representing an estimated probability of receiving the Vicryl mesh protocol was derived from a multivariable logistic regression model incorporating age, race, sex, body mass index, comorbidity index, smoking status, percentage of predicted forced expiratory volume in 1 second (FEV1), percentage of predicted diffusion capacity of the lungs for carbon monoxide (DLCO) divided by alveolar ventilation, hemoglobin level, and albumin level. Within each surgery type (lobectomy or segmentectomy), 1:1 nearest-neighbor matching method without replacement was used, in which a standard patient was matched to Vicryl mesh patient of the same surgery type, provided that the difference in the propensity score was <0.05. To account for the matched pairs, the Wilcoxon signed-rank test or paired *t* test was used to compare the 2 groups for continuous variables, and the McNemar test or random intercept logistic regression analysis was used to compare the 2 groups for categorical variables. The standardized mean difference was calculated for each propensity score parameter to examine the covariate balance between the 2 groups, with a value <0.2 considered small and balanced.^9^ Statistical significance was set at a 2-sided *P* value <.05. Analyses were performed using R version 4.2.3 (R Foundation for Statistical Computing) and SPSS version 22 (IBM).

## Results

### Demographic Information

During the 43-month study period, 250 patients were analyzed, including 125 patients in the mesh group and 125 in the no-mesh group. Demographic data for the 2 cohorts are listed in Table 1. There were no differences in demographics or comorbidities between the 2 groups except for a higher rate of severe chronic obstructive pulmonary disease in the mesh patients. The DLCO/alveolar ventilation percent of predicted was significantly lower in the mesh group, and the Charlson Comorbidity Index was significantly higher (worse) in the mesh cohort. Of interest, preoperative psychotropic drug use was high in both cohorts (36% mesh vs 26% no-mesh).

**Table 1:** Demographic characteristics

Characteristic	Mesh group (N = 125)	No-mesh group (N = 125)	*P* value
Age at operation, y, mean ± SD	68 ± 10	67 ± 9	.4∗
Race, n (%)	–	–	.36†
White	113 (90)	119 (95)	
Black	6 (5)	4 (3)	
Hispanic	5 (4)	1 (1)	
Others	1 (1)	1 (1)	
Sex, n (%)	–	–	.890†
Male	40 (32)	42 (34)	
Female	85 (68)	83 (66)	
BMI, kg/m^2^, mean ± SD	26.7 ± 5.2	26.9 ± 5.5	.9†
Comorbidities, n (%)	–	–	–
COPD, severe	53 (44)	31 (25)	**.005**†
Diabetes	31 (25)	23 (18)	.282†
Heart disease (MI, CHF, angina)	19 (15)	17 (14)	.857†
Hypertension	58 (46)	73 (58)	.076†
Renal insufficiency (CKD ≥III)	9 (7)	7 (6)	.796†
Peripheral vascular disease	11 (9)	8 (6)	.633†
Liver disease	5 (4)	4 (3)	1.000†
Obesity (BMI >30)	29 (23)	30 (24)	1.000†
Connective tissue disease	11 (9)	7 (7)	.463†
Malnutrition (BMI ≤20)	10 (8)	8 (6)	.807†
GI disease, GERD, peptic ulcer disease	39 (31)	31 (25)	.324†
Neurologic/cerebrovascular	4 (3)	3 (2)	1.000†
Prior malignancy‡	57 (46)	67 (54)	.255†
Charlson Comorbidity Index, mean ± SD	6.13 ± 2.11	5.41 ± 2.03	**.002**†
Family history of lung cancer, n, %	33 (26)	30 (24)	.660†
Smoking status, n (%)	–	–	.713†
Current	30 (24)	28 (22)	
Former	71 (57)	77 (62)	
Never	24 (19)	20 (16)	
Pack-years (current or former smokers), mean ± SD	34 ± 23	35 ± 26	>.9∗
Alcohol use, n (%)	–	–	.934†
Daily	28 (22)	27 (22)	
Occasional	65 (52)	66 (53)	
None	32 (27)	29 (23)	
Unknown	0	2 (2)	
Pulmonary function tests, mean ± SD	–	–	
FEV1, L	2.33 ± 0.65	2.38 ± 0.69	.5∗
FEV1% of predicted, mean ± SD	94 ± 18	92 ± 18	.7∗
DLCO/VA % of predicted, mean ± SD	84 ± 18	89 ± 22	**.008**∗
Induction systemic therapy, n (%)	1 (1)	3 (2)	.622§
Preoperative opioid use, n (%)	4 (3)	1 (1)	.370§
Preoperative psychotropic drug use, n (%)	45 (36)	33 (26)	.101†

Bold type indicates statistical significance. *BMI*, Body mass index; *COPD*, chronic obstructive pulmonary disease; *MI*, myocardial infarction; *CHF*, congestive heart failure; *CKD*, chronic kidney disease; *GI*, gastrointestinal; *GERD*, gastroesophageal reflux disease; *FEV1*, forced expiratory volume in 1 second; *DLCO/VA*, diffusion capacity of the lungs for carbon monoxide divided by alveolar ventilation.

∗ Wilcoxon rank-sum test.

† χ^2^ test.

‡ Other than nonmelanoma skin cancer.

§ Fisher exact test.

For the mesh patients, the mean age (32% men, 68% women) was 68 ± 10 years, 19% were never-smokers, and 90% were Caucasian. For the no-mesh patients (34% men, 66% women), the mean age was 67 ± 9 years, 16% were never-smokers, and 95% were Caucasian.

### Surgical Resection

The surgical resection data are presented in Table 2. No significant differences were found between the 2 cohorts. The patients in the mesh cohort underwent 41 lobectomies, 83 segmentectomies, and 25 multilobe procedures, with a mean post-bronchodilator %FEV1 of 94 ± 18 (range, 48-191). Procedures in the no-mesh cohort included 44 lobectomies, 80 segmentectomies, and 21 multilobe procedures, and the mean post-bronchodilator %FEV1 was 92 ± 18 (range, 42-134). Of note, only 8% of mesh patients and 8% of no-mesh patients had benign disease on final pathology. The mean operative time was approximately 2 hours in both cohorts.

**Table 2:** Surgical results

Operative variable	Mesh group (N = 125)	No-mesh group (N = 125)	*P* value
Type of primary resection, n (%)			.35∗
Lobectomy	37 (30)	37 (30)	
Bilobectomy	1 (1)	1 (1)	
Segmentectomy	63 (50)	67 (54)	
Lobectomy plus resection in another lobe	4 (3)	7 (6)	
Segmentectomy plus resection in another lobe	20 (16)	13 (10)	
Operative time, “skin-to-skin”, min, mean ± SD (range)			
All cases	123 ± 27	125 ± 31	.5†
Lobectomy	141 ± 27	146 ± 24	.4†
Segmentectomy	115 ± 23	115 ± 28	.9†
Multilobe procedure	131 ± 33	140 ± 28	.3†
Blood loss, mL, mean ± SD (range)			
All cases	100 ± 225	83 ± 88	.4†
Lobectomy	95 ± 60	85 ± 43	.4†
Segmentectomy	102 ± 267	82 ± 105	.5†
Multilobe procedure	172 ± 451	83 ± 42	.3†
Pathologic tumor stage, n (%)‡		–	.378∗
Stage IA-B	76 (61)	78 (62)	
Stage IIA-B	10 (8)	13 (10)	
Stage IIIA-B	8 (6)	6 (5)	
Multiple primary lung cancers, different lobes	9 (7)	6 (5)	
Stage IV (metastasectomy)	13 (10)	13 (10)	
Pathology type, n (%)			.916∗
Adenocarcinoma	75 (60)	76 (61)	
Squamous cell carcinoma	17 (14)	10 (8)	
Other primary lung carcinoma	6 (5)	6 (5)	
Carcinoid	8 (6)	8 (6)	
Metastases	9 (7)	15 (12)	
Benign (eg, granuloma)	10 (8)	10 (8)	
Tumor size (largest), cm, mean ± SD (range)	2.42 ± 1.76	2.30 ± 1.29	.4§

∗ χ^2^ test.

† Welch 2-sample *t* test.

‡ Total of <225 patients after those with benign resected nodules (9 in each group) were excluded.

§ Wilcoxon rank-sum test.

The hospital cost of the small 6-inch package of Vicryl mesh used in this study is approximately 20% of the cost of 1 hospital day on the surgical nursing floor at our cancer center. The cost of 1 application of Progel lung sealant is approximately 60% of the cost of 1 hospital day on the surgical nursing floor.

### Postoperative Outcomes

The postoperative outcomes for all patients are presented in Table 3. The vast majority of patients in both cohorts did not have postoperative complications (78% for mesh, 82% for no-mesh). There were no empyemas, wound infections, or deaths. There were 2 reoperations in the no-mesh group (1 for air leak and 1 for bleeding) and none in the mesh group. Eight mesh patients and 13 no-mesh patients had an air leak for >2 days. Although numerically longer in the no-mesh cohort, there was no significant difference in mean chest tube duration in days for all resections combined except in the multilobe procedure patients, in whom it was shorter in the mesh cohort (3.21 ± 1.63 days vs 4.67 ± 2.82 days in the no-mesh cohort; *P* = .042). However, more than one-half (55%; n = 69) of the mesh patients had a 1-day duration of chest intubation, compared to only 22% (n = 28) of the mesh patients (*P* = .0001). Three mesh patients with persistent tiny air leaks were discharged with a Heimlich valve on their chest tube, allowing removal of the chest tube later in the clinic. The total number of days the chest tubes remained in place in these 3 patients were included in the chest tube days statistical comparisons.

**Table 3:** Postoperative characteristics

Characteristic	Mesh group (N = 125)	No-mesh group (N = 125)	*P* value
Patients without any complication, n, %	98 (78)	102 (82)	.527∗
Complications, n			
Pneumonia	2	1	1†
Air leak for >2 d	8	13	.254∗
Reoperation for complication (any cause)	0	2	.498†
DVT or PE	0	0	
Bleeding requiring transfusion	0	1	1†
Respiratory insufficiency/BiPAP/reintubation	0	1	1†
Atelectasis requiring bronchoscopy	0	0	
Wound infection	0	0	
Chylothorax	0	1	1†
Empyema or BP fistula	0	0	
Atrial arrhythmia	5	2	.447†
Myocardial infarction	0	0	
Postoperative air leak, d, mean ± SD‡	0.66 ± 2.07	0.81 ± 2.40	.5§
Chest tube duration, d, mean ± SD			
All cases	2.02 ± 2.08	2.38 ± 1.97	.2
Lobectomy	2.31 ± 2.64	3.09 ± 2.58	.2‖
Segmentectomy	1.92 ± 1.77	2.00 ± 1.44	.7‖
Multilobe procedures	2.10 ± 2.08	3.57 ± 3.04	**.065**‖
Chest tube duration 1 d, n (%)	69 (55)	28 (22)	**.0001**‖
Chest tube duration ≥5 d, n (%)	6 (4.8)	10 (8)	.32‖
Hospital stay, d, mean ± SD			
All cases	3.11 ± 1.73	3.62 ± 1.92	**.028**‖
Lobectomy	3.54 ± 2.33	4.39 ± 2.49	.11‖
Segmentectomy	2.93 ± 1.35	3.21 ± 1.38	.2‖
Multilobe procedures	3.21 ± 1.63	4.67 ± 2.82	**.042**‖
Patients with 2-d hospital stay, n (%)	46 (37)	13 (10)	**.0001**‖
Discharge with Heimlich valve, n	3	0	.247†
Mortality, in-hospital and 30-d, n	0	0	
Discharge to rehab or nursing home, n	0	0	
Readmission within 30 d, n (%)	1 (0.8)	0	1†
Complication within 30 d (outpatient), n (%)	5 (4)	2 (2)	.447†
Discharge oral analgesic medication, n (%)			
Acetaminophen	4 (3)	0	.122†
Ibuprofen	4 (3)	0	.122†
Tramadol (<12 MME/d)	44 (35)	47 (38)	.693∗
Hydrocodone (<40 MME/d)	61 (49)	62 (50)	.899∗
Oxycodone (<60 MME/d)	12 (10)	16 (13)	.422∗

Bold type indicates statistical significance.

*DVT,* Deep venous thrombosis; *PE,* pulmonary embolism; *BiPAP,* bilevel positive airway pressure; *BP,* bronchopleural; *MME,* morphine milligram equivalents.

∗ χ^2^ test.

† Fisher exact test.

‡ All types of cases combined.

§ Wilcoxon rank-sum test.

‖ Welch 2-sample *t* test.

The mean hospital length of stay was significantly shorter in the mesh cohort compared to the no-mesh cohort (3.11 ± 1.73 days vs 3.62 ± 1.92 days; *P* = .028). The decrease in hospital length of stay was most notable in patients undergoing a multilobe procedure in the mesh cohort versus the no-mesh cohort (3.62 ± 1.92 days vs 4.67 ± 2.82 days; *P* = .042). However, well over one-third of mesh patients (37%; n = 46) had just a 2-day hospital stay, compared to 10% (n = 13) of no-mesh patients. There was 1 readmission for atrial fibrillation within 30 days in the mesh group. The rate of outpatient complications was very low in both groups (4% for mesh; 2% for no-mesh), and the most frequent complication was a sterile serous pleural effusion necessitating therapeutic thoracentesis. Virtually all of the patients in both cohorts (10% mesh, 13% no-mesh) who required oxycodone (>60 morphine milligram equivalent/day) at hospital discharge had a preoperative nonmalignant chronic pain syndrome requiring opioids.

The propensity score–matched analysis yielded 84 matched pairs (n = 168) from a total of all 252 patients. Refining the propensity score matching approach to match patients within the same surgery type (lobectomy and segmentectomy), resulted in 79 pairs. Both the hospital length of stay and duration of chest intubation were significantly shorter in the mesh group (Table 4). Although numerically the days of postoperative air leak was lower in the mesh group, the difference was not statistically different.

**Table 4:** Propensity score–matched lobectomy and segmentectomy (combined) postoperative results

Surgery type	Characteristic	Mesh group (N = 79)	No-mesh group (N = 79)	*P* value∗
All	Postoperative air leak, d, mean ± SD	0.47 ± 1.45	0.68 ± 2.19	.449
All	Chest tube duration, d, mean ± SD	1.77 ± 1.41	2.32 ± 1.82	**.028**
All	Hospital stay, d, mean ± SD	2.91 ± 1.08	3.53 ± 1.69	**.004**
All	30-d complications, n (%)	4 (5.1)	1 (1.3)	.371

Bold type indicates statistical significance.

∗ Paired *t* test; McNemar χ^2^ test with continuity correction.

## Discussion

Despite steady advances in the operative approach to lung resection, the issue of postoperative air leak persists, occurring in 30% to 58% of cases.^1^^,^^2^ More than 15% of patients will have a long-term air leak over 7 days, often leading to increased postoperative complications and always to a prolonged hospital stay.^10^ Numerous intraoperative and postoperative strategies have been proposed to prevent air leaks, with varying degrees of success^3^ (Table 5).

**Table 5:** Published techniques to achieve pneumostasis and early chest tube removal

1. Release of the inferior pulmonary ligament and any adhesions to mobilize the lung superiorly to reduce air space for more pleural–pleural approximation 2. Use of fissure-less dissection technique 3. Creation of apical parietal pleural tent for upper lobe resections 4. Creation of pneumoperitoneum to elevate the diaphragm for lower lobectomies and bilobectomies 5. Careful suturing of air leaks with absorbable suture buttressed with muscle or felt pledgets 6. Intercostal muscle flap coverage of repaired staple or suture line 7. Chemical lung sealants: Fibrin glue (Tisseel), Bioglue, and Progel 8. Peri-Strip pericardial stapler sleeves to buttress the staple lines 9. Digital drainage systems to quantify air leaks to facilitate early chest tube removal (Thopaz and Thoraguard systems) 10. Intrapleural chemical pleurodesis 11. Autologous blood patch 12. Low suction on the chest tube or water seal only 13. Transbronchoscopic placement of endobronchial valves 14. Discharge with chest tube and Heimlich valve for later outpatient chest tube removal 15. Reoperation to repair air leak with/without intercostal muscle flap coverage of the leak 16. Polyglycolic mesh (Vicryl) and chemical lung sealant (Progel), current series

Most air leaks result from a communication of the lung parenchyma to the pleural space distal to a segmental bronchus (alveolar-pleural fistula) and are distinct from the very uncommon bronchopleural fistula.^11^ Cerfolio described a classification system of 4 types of air leaks: forced expiratory leaks (occurs with a cough only), expiratory leak, inspiratory air leak, and continuous air leak.^11^ Because most lung resections use stapling devices to separate lung tissue, air leaks usually emanate from the parenchymal staple line(s).

Risk factors for a postoperative air leak include chronic obstructive pulmonary disease with emphysematous lungs, which is the most consistent factor, with a reduced FEV1 <70% is the most important factor.^12^ Other risk factors include chronic steroid use, smoking history, male sex, pleural adhesions, decreased DLCO, increased body mass index, and poor nutritional status. Surgical procedures at highest risk for a postoperative air leak include lobectomy more than wedge resection or segmentectomy, and especially an upper lobectomy and bilobectomy.^3^ An air leak lasting ≥5 days is classified as prolonged. Prolonged air leak was reported to occur in 10.4% of patients who underwent lung resection in the Society of Thoracic Surgeons General Thoracic Database cohort of 50,000 lung resection patients.^13^

### Polyglycolic Acid Mesh Experience

In 2007, Ueda and colleagues^4^ published a small series of 45 patients undergoing thoracoscopic lung resections. Twenty-eight of these patients had an intraoperative proven air leak and underwent suture repair of the leak, followed by application of polyglycolic acid mesh to cover the parenchymal suture line, held in place with fibrin glue. This method successfully stopped air leaks in these 28 patients in a mean of 1 day, although these patients had a mean hospital length of stay of 7.1 days. The authors reported that their experience adding the mesh to the fibrin glue technique worked far better than their prior approach of using fibrin glue only to achieve pneumostasis.^14^

Subsequently Murakami and colleagues^15^ reported their experience with thoracoscopic lung resection in which 112 of their 162 patients (69%) had an intraoperative air leak when tested in the saline-filled chest. They placed polyglycolic mesh held in place with fibrin glue over the parenchymal staple lines. Their experience with this technique was also favorable, with a mean duration of chest tube drainage 1.1 ± 2.6 days. However, the authors did not include a control cohort, which weakened this study.

### Current Study

Based on the experience of Ueda and colleagues^4^ and Murakami and colleagues,^15^ we elected to add this technique of using a double-layer of polyglycolic mesh over the parenchymal staple lines for all consecutive major lung resections (segmentectomies, lobectomies and multilobe resections) to see if we had improved pneumostasis. After any air leaks found with pressure testing were repaired just prior to chest closure, the mesh was placed over the parenchymal staple lines and held in place with Progel lung sealant. We retrospectively compared the outcomes of the consecutive series of mesh patients to those in a consecutive control cohort of patients who underwent the same resections with the same techniques except for use of the mesh. As in previous studies, we also found a significantly shorter time to chest tube removal and a significantly decreased hospital length of stay (Table 3) without any perioperative morbidity. The results were most pronounced in multilobe resections, as expected, because there are more parenchymal staple lines in this operation. This simple technique takes only 4 to 5 minutes, and the cost of extra material is low, less than that in our control cohort because we needed to use only 1 vial of the more expensive Progel instead of the usual 2 vials.

### Previously Published Techniques for Pneumostasis

Various intraoperative surgical strategies to prevent air leaks have been proposed. Increasing the visceral-to-parietal pleural apposition is a key strategy when possible. Without a large postoperative intrapleural air space, air leaks will be less frequent. Releasing the inferior pulmonary ligament and lysis of all intrapleural adhesions will help mobilize the lung.^16^ In patients with fused fissures, use of the fissureless technique is advocated when the lung parenchyma is divided with surgical staplers after transection of the lobar bronchus.^17^ Creation of an apical pleural tent allows the apical parietal pleura to drape over the apex of the lung and may help seal air leaks with upper lobectomies and apical segmentectomies.^18^^,^^19^ For right middle and right lower lobe lobectomies (bilobectomy), creation of a pneumoperitoneum to elevate the diaphragm superiorly to decrease the intrapleural space may successfully reduce any air leak and has been described but is not used routinely.^20^^,^^21^ Multiple studies have been published on the use of intraoperative surgical sealants such as Progel to decrease parenchymal air leaks, with mildly positive findings resulting in a median decrease in hospital length of stay of 1.5 days, as discussed in a meta-analysis of published studies.^22^ The advent of lung volume reduction surgery for severely emphysematous lungs and the associated major postoperative air leaks led to the development of premade pericardial sleeves (Peri-Strips; Baxter International), which are placed over the jaws of the lung stapler to buttress the staple lines in these patients with or without the use of Bioglue, with a resulting reduction in air leaks.^23^ Currently, however, Peri-Strips are used mainly in lung volume reduction surgery and in bariatric and other types of gastrointestinal surgery.

There are numerous available postoperative interventions for handling air leaks. Sophisticated digital systems that quantify air leaks, such as the Thopaz and Thoraguard systems, are used by some providers to determine when to remove chest tubes. Digital systems allow for continuous interpretation of data trends and identifying intermittent air leaks. In prospective trials, digital drainage systems have reduced the duration of chest intubation and hospital length of stay compared to standard analog pleural drainage systems.^24^

Other interventional methods that have been used with varying degrees of success to resolve prolonged air leak include chemical pleurodesis,^25^ autologous blood patches,^26^ transbronchoscopic placement of endobronchial valves,^27^ suction versus no suction on the underwater seal drains,^28^ discharge to home with a chest tube in place with a Heimlich valve (Pneumostat; Atrium Medical) on the tube for eventual tube removal in the clinic,^29^ and reoperation to repair the air leak, possibly with an intercostal muscle flap to cover the leaking site.^12^

Even with the aforementioned techniques, postoperative air leaks still occur, particularly in patients with obvious emphysematous lungs and reduced pulmonary function. Based on our experience and prior studies, we feel that thoracic surgeons will find that selectively implanting a double layer of polyglycolic mesh over the parenchymal staple line(s) is a quick, safe and inexpensive adjunct to achieve pneumostasis with no significant drawbacks, and which is particularly useful in patients with challenging anatomic findings.

## Limitations

Retrospective case reviews including “before-after” designs such as the present study have some intrinsic limitations, including potential selection bias, which might confound the results. However, because the 2 cohorts are consecutive patient series, and all patients in the polyglycolic acid mesh cohort underwent the same surgical procedures using exactly the same techniques as the control cohort, selection bias is minimized. Strengths of the study include the large number of patients with 2 balanced cohorts differing only in the use of mesh, and the fact that all operations were performed by the same surgeon using the same surgical techniques and postoperative care protocol. Nonetheless, these single-surgeon results might not be generalizable to all surgical practices.

In addition, all surgical procedures were performed using a limited open thoracotomy, but the technique can be readily applied to a minimally invasive surgical approach, as demonstrated by the earlier published studies, which were all thoracoscopic lung resections. Finally, because the surgeon (L.A.R.) already has been routinely using many of the previously published techniques to enhance pneumostasis with good results and short hospital stays, adding the double-layer mesh technique significantly improved the results, but differences in the cohorts were not as striking as they would have been had he used only standard techniques in the control cohort. One of the coauthors (S.F.) is a thoracic surgeon who exclusively uses a robotic-assisted approach to lung resection. He routinely uses the double-layer mesh technique, finding it easy to use and a quite effective adjunct to pneumostasis.

## Conclusions

Regardless of the operative approach or the surgeon's experience, postoperative air leaks remain a common complication, occurring in as many as one-half of lung resections, and will persist for >5 days in at least 10% of patients. Although numerous techniques to improve pneumostasis have been described, we found that placing a double layer of polyglycolic acid mesh over the parenchymal staple line(s) at the end of a major lung resection is a safe, quick, and effective adjunct to reduce postoperative air leaks. This procedure results in a significant decrease in hospital stay for patients with major lung resections, especially multilobe procedures, which have more staple lines.

## Conflict of Interest Statement

The authors reported no conflicts of interest.

The *Journal* policy requires editors and reviewers to disclose conflicts of interest and to decline handling or reviewing manuscripts for which they may have a conflict of interest. The editors and reviewers of this article have no conflicts of interest.
